# Wood Ash Induced pH Changes Strongly Affect Soil Bacterial Numbers and Community Composition

**DOI:** 10.3389/fmicb.2017.01400

**Published:** 2017-07-28

**Authors:** Toke Bang-Andreasen, Jeppe T. Nielsen, Jana Voriskova, Janine Heise, Regin Rønn, Rasmus Kjøller, Hans C. B. Hansen, Carsten S. Jacobsen

**Affiliations:** ^1^Department of Environmental Science, Aarhus University Roskilde, Denmark; ^2^Department of Biology, University of Copenhagen Copenhagen, Denmark; ^3^Department of Geochemistry, Geological Survey of Denmark and Greenland (GEUS) Copenhagen, Denmark; ^4^Department of Plant and Environmental Sciences, University of Copenhagen Frederiksberg, Denmark; ^5^Ecology Department, Climate and Ecosystem Sciences, Lawrence Berkeley National Laboratory, Berkeley CA, United States; ^6^Center for Permafrost (CENPERM), University of Copenhagen Copenhagen, Denmark; ^7^Section for Geomicrobiology, GFZ German Research Centre for Geosciences Potsdam, Germany; ^8^Key Laboratory of Urban Environment and Health, Institute of Urban Environment, Chinese Academy of Sciences Xiamen, China; ^9^Arctic Station, University of Copenhagen Qeqertarsuaq, Greenland

**Keywords:** wood ash, renewable energy, forest soil, bacterial community, bacterial numbers, 16S rRNA, CFU, biodiversity

## Abstract

Recirculation of wood ash from energy production to forest soil improves the sustainability of this energy production form as recycled wood ash contains nutrients that otherwise would be lost at harvest. In addition, wood-ash is beneficial to many soils due to its inherent acid-neutralizing capabilities. However, wood ash has several ecosystem-perturbing effects like increased soil pH and pore water electrical conductivity both known to strongly impact soil bacterial numbers and community composition. Studies investigating soil bacterial community responses to wood ash application remain sparse and the available results are ambiguous and remain at a general taxonomic level. Here we investigate the response of bacterial communities in a spruce forest soil to wood ash addition corresponding to 0, 5, 22, and 167 t wood ash ha^-1^. We used culture-based enumerations of general bacteria, *Pseudomonas* and sporeforming bacteria combined with 16S rRNA gene amplicon sequencing to valuate soil bacterial responses to wood ash application. Results showed that wood ash addition strongly increased soil pH and electrical conductivity. Soil pH increased from acidic through neutral at 22 t ha^-1^ to alkaline at 167 t ha^-1^. Bacterial numbers significantly increased up to a wood ash dose of 22 t ha^-1^ followed by significant decrease at 167 t ha^-1^ wood ash. The soil bacterial community composition changed after wood ash application with copiotrophic bacteria responding positively up to a wood ash dose of 22 t ha^-1^ while the adverse effect was seen for oligotrophic bacteria. Marked changes in bacterial community composition occurred at a wood ash dose of 167 t ha^-1^ with a single alkaliphilic genus dominating. Additionally, spore-formers became abundant at an ash dose of 167 t ha^-1^ whereas this was not the case at lower ash doses. Lastly, bacterial richness and diversity strongly decreased with increasing amount of wood ash applied. All of the observed bacterial responses can be directly explained by the wood ash induced changes in pH, electrical conductivity and the addition of wood ash inherent nutrients.

## Introduction

The increasing use of wood for energy production leads to increased production of wood ash (Karltun et al., 2008; Huotari et al., 2015). Wood ash is often treated as a waste product due to its content of toxic elements (e.g., Cd, As, Cr, and Ni) and a large proportion of the produced wood ash is therefore deposited in landfills (Vance, 1996; Demeyer et al., 2001). This leads to considerable loss of valuable plant nutrients and potential acidification of forest plantation ecosystems (Olsson et al., 1996; Augusto et al., 2008). Wood ash retains most of the major mineral plant nutrients except nitrogen and has liming properties owing to its high content of metal oxides and hydroxides (Demeyer et al., 2001; Augusto et al., 2008). Recirculation of wood ash to forest soils can thus return valuable nutrients to forest ecosystems and counteract soil acidification making energy production by combustion of wood more sustainable.

Wood ash is highly reactive in soil (Karltun et al., 2008) and alters several physio-chemical properties of the soil. Hence, addition of wood ash leads to an increase in soil pH and pore water electrical conductivity and increased concentrations of elements such as the nutrients K, S, B, Na, Ca, Mg, Si, Fe, and P (Ohno and Susan Erich, 1990; Demeyer et al., 2001; Pitman, 2006; Augusto et al., 2008). The increase in pH also changes bioavailability of soil nutrients due to pH-dependent soil chemical equilibria (Khanna et al., 1994; Demeyer et al., 2001). Soil microorganisms are known to respond to these wood ash induced changes (Aronsson and Ekelund, 2004; Huotari et al., 2015). In particular soil pH (Fierer and Jackson, 2006; Lauber et al., 2009; Rousk et al., 2010; Kaiser et al., 2016; Kim et al., 2016) and electrical conductivity (which is correlated to pore water salt concentration) (Lozupone and Knight, 2007; Kim et al., 2016) have been shown to be main drivers in shaping bacterial communities in various soils. Additionally, marked changes to soil systems are known to induce sporulation of soil bacteria as a survival mechanism to unfavorable conditions (Lowe et al., 1989; Nicholson et al., 2000).

Soil bacteria are essential forest soil ecosystem drivers carrying out processes such as decomposition of organic matter, nutrient cycling and greenhouse gas production and play a crucial role as part of the lower trophic levels of the soil food web (Paul, 2014). Changes in bacterial community can thus affect soil functioning and soil quality (Wittebolle et al., 2009; Philippot et al., 2013). Indirect measures of wood ash effects on soil bacteria have detected increases in overall mineralization- (Zimmermann and Frey, 2002; Björk et al., 2010; Saarsalmi et al., 2010, 2012), decomposition- (Weber et al., 1985; Moilanen et al., 2002) and respiration rates (Bååth and Arnebrant, 1994; Fritze et al., 2000; Perkiömäki and Fritze, 2002; Zimmermann and Frey, 2002; Perkiömäki et al., 2003). Direct bacterial responses to wood ash addition include changes in bacterial numbers measured by colony forming units (CFU) (Bååth and Arnebrant, 1994; Fritze et al., 2000), bacterial growth rates measured by ^3^H-thymidine incorporation (Bååth and Arnebrant, 1994; Fritze et al., 2000; Perkiömäki and Fritze, 2002), bacterial community structures analyzed using PLFAs (Frostegård et al., 1993; Fritze et al., 2000; Liiri et al., 2002; Perkiömäki and Fritze, 2002; Mahmood et al., 2003; Peltoniemi et al., 2016; Cruz-Paredes et al., 2017), 16S rRNA DGGE (Perkiömäki and Fritze, 2003), T-RFLP (Noyce et al., 2016) and amplicon sequencing (Noyce et al., 2016) and bacterial substrate utilization using Biolog plates (Fritze et al., 2000; Merino et al., 2016). The majority of these studies report increased bacterial growth rates and quantities after wood ash addition combined with changes in bacterial community structure and function. These changes are correlated to the applied dose of wood ash and have generally been linked to ash induced pH increases in soil. Despite of this, reports on bacterial responses to ash addition remain relatively sparse and ambiguous (Aronsson and Ekelund, 2004). Additionally, the vast majority of reported bacterial community responses are the results of methods that only reveal shifts in bacterial community at a general taxonomic level (e.g., PLFA, DGGE, and T-RFLP). More knowledge on bacterial responses at lower taxonomic levels is thus strongly needed to reveal wood ash induced responses in specific bacterial groups which are important for soil ecosystem functioning.

In our study, we aim to investigate bacterial community responses to wood ash addition in a spruce forest soil using 16S amplicon sequencing of bacterial rDNA and three CFU approaches. The high-throughput sequencing technique allows an in-depth analysis of bacterial communities. We investigate the application of wood ash in doses within the current legislation (5 t ha^-1^), four times the currently allowed dose (22 t ha^-1^) and at an extreme dose of 167 t ha^-1^. The extreme dose was included to allow us to investigate if high ash doses will tip the system and induce detrimental effects on the soil bacterial community. Furthermore, because we hypothesize that wood ash induced changes in soil pH will be a main driver in shaping the soil bacterial community the chosen wood ash doses represent an increasing soil pH from acidic through neutral to alkaline conditions.

We hypothesize that changes in pH and salinity induced by wood ash application will cause significant changes in the soil bacterial community and that these community changes can be observed gradually over time. We further hypothesize that bacterial numbers (CFUs) initially will be stimulated with increasing wood ash dose as the pH of the acidic forest soil will increase until a maximum over which the bacterial abundance decreases due to an alkaline pH or because other wood ash derived properties become harmful for the majority of soil bacteria. Finally, we also hypothesize that the application of wood ash in high doses will cause unfavorable conditions for most of the indigenous bacteria which are adapted to the non-manipulated soil resulting in a larger frequency of spore forming bacteria.

## Materials and Methods

### Soil and Wood Ash

Soil was collected in May 2014 from “Gedhus Plantage” (56°16′38″N, 09°05′12″E) a 57-year-old, 2nd generation Norway spruce [*Picea abies* (L.) Karst.] plantation situated in a relatively undisturbed (i.e., no tilling or addition of fertilizers) previous heathland. The climate is temperate with mean annual precipitation of 850 mm and mean annual temperature of 8.4°C. The soil, classified as a podzol, was collected from the O-horizon (0–10 cm) and homogenized by first removing large roots followed by sieving (4 mm mesh).

The wood ash was a mixture of bottom- and fly ash collected from the local Brande heating plant produced by combustion of wood chips from predominantly coniferous trees. The ash was homogenized by sieving (2 mm mesh).

Properties of the soil and wood ash are presented in **Table 1**. Additional elemental composition of the soil and the wood ash can be further inspected in Qin et al. (2017) where the soil and the ash used in the present study were named “Gedhus soil” and “Brande Ash,” respectively.

**Table 1:** Properties of soil and wood ash.

	WC (%)	OM (%)	pH	Conductivity (μS cm^-1^)
Soil:	145.3 ± 0.5	80.5 ± 0.3	4.33 ± 0.03	108.2 ± 12.0
Wood ash:	0.60 ± 0.01	6.50 ± 0.2	12.9	20,400


*“WC (%)” refers to gravimetric water content (g water g^-*1*^ dry weight soil). “OM (%)” refers to organic matter (loss on ignition – g organic matter g^-*1*^ dry weight soil). “±” denote standard error of the mean (SEM) of triplicates. Numbers without “±” are measurement on one replicate.*

### Microcosms

The homogenized soil and wood ash were separately and repeatedly processed through a *riffle splitter* to acquire sufficient representative samples. Microcosms were established in triplicates with 150 g of soil in 1 l screw cap glass bottles with wood ash added in doses of 0, 5, 22, and 167 t ha^-1^ (see Supplementary Information 1 for wood ash concentration calculations). The ash doses were chosen based on pilot experiments showing that this range in dosages resulted in pH responses in the soil ranging from acidic through neutral to alkaline. Additionally, the 5 t ha^-1^ wood ash dose corresponds approximately to the upper limit of what is currently allowed to add to forest soils in Scandinavian countries (Huotari et al., 2015) while the doses of 22 and 167 t ha^-1^ were included to test for bacterial responses in the soil with doses above the current legislation threshold. Water was added to the microcosms corresponding to 50% of soil water holding capacity of the soil and ash was mixed well to achieve a homogenous mixture. Microcosms were incubated at 10°C under dark and aerobic conditions for 42 days and soil samples were aseptically collected from the microcosms throughout the incubation period to analyze changes in pH, electrical conductivity, and the bacterial community using cultivation-based approaches and 16S rRNA gene amplicon sequencing, as described below.

### Electrical Conductivity and pH

Electrical conductivity and pH were measured 1, 3, 7, 15, 28, and 42 days after start of incubation. Soil slurries of 5 g soil to 25 ml Milli-Q water were shaken for 1 h followed by sedimentation for 2 h. Afterward electrical conductivity was measured in the supernatant using a TetraCon 325 electrode adapted to a conductivity meter Cond 340i (WTW, Weilheim, Germany) followed by measurements of pH using Sentix 940 electrode connected to a pH meter Multi 9310 (WTW).

### Cultivation of Bacteria

Soil for estimation of numbers of CFU was sampled from the microcosms 3 days after start of incubation. From each microcosm 0.1 g of soil was collected and used to generate a dilution series in 1X phosphate buffered saline (PBS) (pH 7.4) ranging from 10^-1^ to 10^-5^. Subsamples of 50 μl of relevant soil dilutions were plated on agar media to quantify (i) general bacteria, (ii) spore forming bacteria and (iii) *Pseudomonas*.

(i) The growth medium for general bacteria was prepared by combining 7.5 g Difco^TM^ Agar, granulated (Becton, Dickinson and Company, Franklin Lakes, NJ, United States) as the solidifying agent, 0.3 g Tryptic Soy Broth (TSB) (MoBio, Carlsbad, CA, United States) as the cultivation medium and added Milli-Q water up to a final volume of 500 ml. The media was autoclaved at 121°C for 20 min and left to cool to 50°C before adding 1 ml anti-fungi agent Delvo-Cid (100 mg l^-1^ in final solution; DSM Food Specialties, Heerlen, Netherlands).

(ii) The growth medium for the spore forming bacteria was prepared as described above only modified by using 1.5 g of TSB (MoBio). The soil dilutions used on this medium were pasteurized, i.e., heated to 80°C for 10 min and cooled to room temperature before inoculation on the TSA in order to largely eliminate vegetative bacteria and thus selecting for spore forming bacteria (Vieira and Nahas, 2005).

(iii) The Gould’s S1 medium (Gould et al., 1985), selective for *Pseudomonas*, was prepared by combining 9 g Difco^TM^ Agar (Becton, Dickinson and Company), 5 g sucrose, 5 ml glycerol, 2.5 g casamino acids (Becton, Dickinson and Company), 0.5 g NaHCO_3_, 0.5 g MgSO_4_⋅7H_2_O, 1.15 g K_2_HPO_4_, 0.6 g sodium lauroyl sarcosine (293.4 M), 20 mg trimethoprim (Sigma–Aldrich, St. Louis, MO, United States) and added Milli-Q water up to a volume of 500 ml.

Dilutions of all samples were inoculated on the different media in five drops of 10 μl and were incubated for 7 days at 10°C. CFUs were quantified by visual counts of colonies on a CK40 stereo microscope (Olympus, Japan).

### DNA Extraction and Sequencing Library Preparation

DNA was extracted from 0.25 g of soil collected from each microcosm 1, 3, 7, 15, 28, and 42 days after start of incubation (in total 72 samples) using PowerLyzer^TM^ PowerSoil^®^ DNA Isolation Kit (MoBio). A NanoDrop ND-1000 spectrophotometer (Thermo Fischer Scientific Inc., Waltham, MA, United States) was used to measure DNA concentration and to verify DNA purity.

For amplifying the bacterial community from the soil samples the primer pair 515f/806r targeting V4 region of 16S rRNA gene was used [515f: 5′-GTGCCAGCMGCCGCGGTAA-3′; 806r: 5′-GGACTACHVGGGTWTCTAAT-3′; (Caporaso et al., 2012)]. PCR amplifications were performed with primers containing template-specific sequences extended by 2-nt linker and 4-6-nt barcode (sequence nucleotides of the barcode system are listed in Supplementary Table 1). Each of three independent 10 μl reactions per DNA sample contained 2 μl 5x PCRBIO Reaction Buffer (PCR Biosystems Ltd., London, United Kingdom), 1 μl Bovine Serum Albumin (BSA) (Bioron, Ludwigshafen, Germany), 0.2 μl dNTP (10 mM), 0.5 μL of each primer (10 μM each) with sample specific barcode combinations, 0.1 μl PCRBIO HIFI Polymerase (PCR Biosystems Ltd., London, United Kingdom), 0.5 μl DNA template and 5.2 μl ddH_2_O. The cycling conditions were as follows: 95°C for 1 min, followed by 30 cycles at 95°C for 15 s, 50°C for 20 s, then 72°C for 20 s, 72°C for 5 min. The resulting amplicons from technical triplicates were pooled, and amplification was verified by agarose gel electrophoresis. Amplicons were purified using HighPrep^TM^ PCR clean up system (Magbio Genomics, Gaithersburg, MD, United States), DNA concentration was quantified by Qubit^®^ HS DNA assay (Life Technologies, Darmstadt, Germany) and samples were equimolarly pooled.

Ligation of Illumina adapters was performed using TruSeq DNA PCR-Free LT Sample Prep Kit (Illumina, San Diego, CA, United States). The final amplicon library was subjected to sequencing on Illumina MiSeq 2 × 250 bp pair-end platform at the National High-throughput DNA Sequencing Centre (Copenhagen, Denmark).

### Bioinformatics

Sequences (deposited in GenBank, accession numbers SAMN06628931 – SAMN06629002) were processed and analyzed using the following pipeline: Paired-end reads were merged using PEAR (Zhang et al., 2014) with minimum overlap size set to 45 bp. Demultiplexing and quality filtering were carried out using QIIME 1.9.1 (Caporaso et al., 2010b) with the following quality filtering settings: Maximum number of consecutive low quality base calls was 5, removal of reads with average Phred quality score below 25, minimum and maximum sequence length was 200 and 320, respectively, maximum number of homopolymers was 6 and sequences that contained ambiguous nucleotides were removed. Chimeras were removed using ChimeraSlayer with the “Gold” reference database (Haas et al., 2011). Operational taxonomic units (OTUs) were picked *de novo* using UCHIME (Edgar et al., 2011) with <97% sequence similarity to separate OTUs. Centroid sequences for each OTU were determined using UCLUST (Edgar, 2010) followed by alignment of sequences using PyNAST (Caporaso et al., 2010a) and taxonomy assignment using UCLUST against the Greengenes database (v. 13_8) (DeSantis et al., 2006). An approximately maximum-likelihood phylogenetic tree was constructed from the centroid sequences using FastTree (Price et al., 2010).

R (v. 3.3.1) (R Core Team, 2017) and the packages vegan (Oksanen et al., 2008) and Phyloseq (McMurdie and Holmes, 2013) were used for further processing, analysis and graphical visualization of the OTU table after removal of singletons, as described in the following.

Metrics of richness (number of observed OTUs) and diversity (Shannon diversity) were calculated based on a rarified number of sequences per sample. Rarefication was done to compensate for variation in read numbers across samples (limit set from the sample with the lowest number of sequences: 10,527 sequences).

The OTU table was normalized using DeSeq2 (Love et al., 2014) prior to calculation of beta-diversity distance matrix using weighted UniFrac metric. The weighted UniFrac dissimilarity matrix was plotted using principal coordinates analysis (PCoA).

### Statistical Analysis

Two-way analysis of variance (ANOVA) was used to test for the effect of ash dose and incubation time on soil pH, electrical conductivity, observed OTUs and Shannon diversity. Because interactions were present in all cases one-way ANOVAs were conducted within each ash dose to test for significant changes in the measurements listed above with incubation time only. One-way ANOVAs were used to test for significant difference in CFUs at the different wood ash doses both within and between the three bacterial groups examined (general bacteria, sporeforming bacteria, and *Pseudomonas*). The following applies to all the above described ANOVAs: (i) Shapiro–Wilk test was used to test for normal distribution of data. (ii) Levenes test was used to test for equality of variances prior to running ANOVA’s and square root data transformation was used when Levenes test showed significant different variances (only true for CFU results). (iii) All ANOVA’s were followed by *post hoc* tests of pair-wise comparisons with Tukey’s honest significant difference method.

Linear Pearson correlations were made to test for correlations between wood ash concentrations, soil pH and soil conductivity. Pearson correlation was also used to test for correlations between wood ash concentration, pH and soil conductivity to observed OTUs and Shannon diversity.

White’s non-parametric *t*-test was used to test for significant differences in relative abundance within the bacterial groups of *Alkalibacterium*, *Paenibacillus*, and *Pseudomonadaceae*. These tests were carried out between day 1 and the remaining incubation times within each of the bacterial groups and within each ash dose. *p*-values were adjusted for false discovery rate using the Benjamini–Hochberg method. The software Statistical Analyses of Metagenomic Profiles (STAMP) (Parks and Beiko, 2010) was used to conduct these tests.

Adonis (PERMANOVA) on weighted UniFrac dissimilarity matrix was used to test for significant differences in community composition with the variables ash concentration, pH, electrical conductivity and incubation time. The vegan function Betadisper was used to test for homogeneity of group dispersions.

## Results

### pH and Electrical Conductivity

**FIGURE 1:**
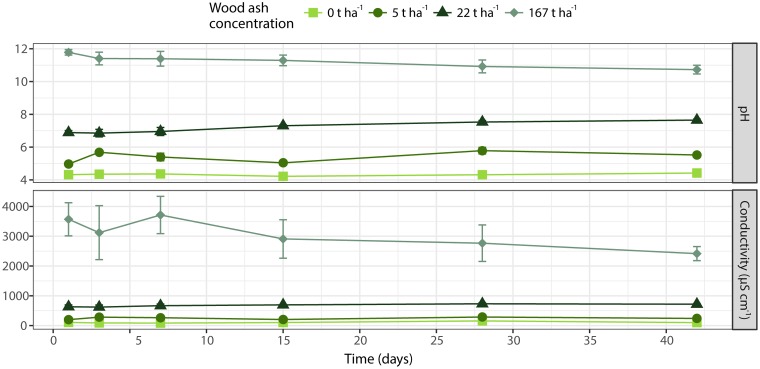
Soil pH **(top)** and electrical conductivity **(bottom)** during incubation time. Different wood ash concentrations are represented with different colors and symbols. Each symbol denotes average of triplicates ± SEM.

Wood ash application was positively correlated to soil pH (*r* = 0.95, *p* < 0.001) and electrical conductivity (*r* = 0.92, *p* < 0.001) (**Figure 1**). Soil pH was positively correlated to electrical conductivity (*r* = 0.98, *p* < 0.001). Incubation time had no significant effect on pH (*p* = 0.856) and electrical conductivity (*p* = 0.767). However, there was a significant (*p* = 0.003) interaction between time and ash concentration on pH as shown by a significant increase in pH with time at wood ash doses 5 t ha^-1^ (*p* = 0.016) and 22 t ha^-1^ (*p* = 0.011) whereas time had no effect at wood ash doses 0 and 167 t ha^-1^. No significant (*p* > 0.05) interaction between time and ash concentration on soil electrical conductivity was observed.

### Culturable Bacteria

**FIGURE 2:**
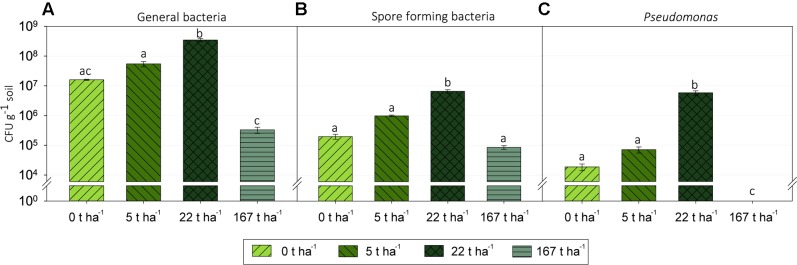
Colony forming units (CFU) per gram of soil in the microcosms of increasing wood ash concentration at day 3 of incubation on agar media selecting for: **(A)** General bacteria, **(B)** spore forming bacteria and **(C)** *Pseudomonas* spp. Statistically significant effect of wood ash addition on CFU counts (*p* < 0.05) within each media type is indicated by different letters. Bars represent average CFU g^-1^ soil of three replicates ± SEM. Detection limit for the general bacteria and the spore forming bacteria was 200 CFU g^-1^ soil and 400 CFU g^-1^ soil for the Pseudomonas selective media.

Wood ash addition significantly (*p* < 0.05) affected colony-forming units (CFU) of the three bacterial groups examined 3 days after start of incubation (**Figure 2**). All changes in CFU followed the same pattern for all three bacterial groups with significant increases (*p* < 0.05) in CFU from ash dose 0 t ha^-1^ up to 22 t ha^-1^ followed by significant decreases (*p* < 0.05) in CFU from wood ash dose 22 t ha^-1^–167 t ha^-1^. CFU counts revealed that there were significant (*p* < 0.001) less spore forming bacteria than general bacteria in the soil at ash dose 0, 5, and 22 t ha^-1^ while no significant (*p* > 0.05) differences were seen at ash dose 167 t ha^-1^. Ratios of spore forming bacteria to general bacteria in the different ash concentrations are shown in Supplementary Figure 1.

### Bacterial Community Sequences

A total of 2,139,860 reads remained after quality filtering and removal of chimeras and singletons with 10,527 to 48,759 sequences per sample. A total of 69,690 unique OTUs were obtained from these sequences.

#### Richness and Diversity

**FIGURE 3:**
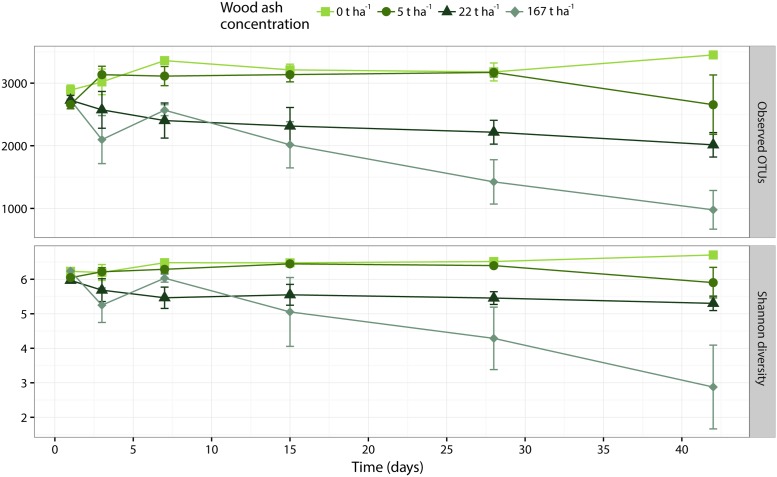
Alpha diversity metrics of number of observed OTUs **(top)** and Shannon diversity **(bottom)** plotted against incubation time. Different colors and shapes represent different added wood ash concentrations. Each point represents average of triplicates ± SEM.

Operational taxonomic unit richness (number of OTUs) and Shannon diversity significantly (*p* < 0.001 for both metrics) decreased with increasing wood ash doses (**Figure 3**). The interaction between ash dose and time was significant for both metrics (observed OTUs: *p* = 0.002; Shannon diversity: *p* = 0.015) and therefore we performed one-way ANOVA followed by Tukey analysis within each ash concentrations to test for significant effects of time on richness and diversity. At 167 t ha^-1^ ash concentration the number of observed OTUs significantly (*p* = 0.009) decreased with time while Shannon diversity showed a trend (*p* = 0.082) to decrease during incubation. The Tukey test revealed that these decreases were present when comparing incubation times of 1 and 7 days against 42 days. At ash dose 0 t ha^-1^ the two metrics significantly (observed OTUs: *p* = 0.042; Shannon diversity: *p* = 0.044) increased during incubation. The Tukey test showed that these significances were present only when comparing days 1–42 of incubation. Both metrics did not change significantly (*p* > 0.05) during incubation at wood ash concentration 5 and 22 t ha^-1^.

**FIGURE 4:**
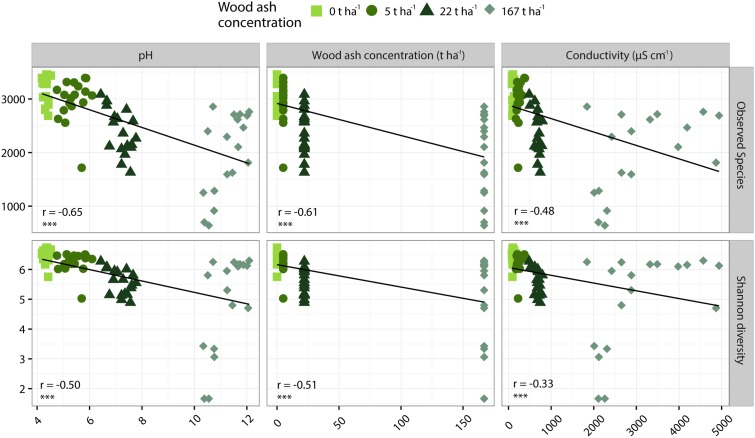
Pearson correlations of number of OTUs **(top row)** and Shannon diversity **(bottom row)** to soil pH **(left column)**, wood ash concentration **(middle column)** and electrical conductivity **(right column)**. *r-* and *p*-values of each Pearson correlation is given in lower left corner of each plot with *p* < 0.001 indicated by ^∗∗∗^.

Richness and Shannon diversity were significantly negatively correlated to wood ash concentration, pH and electrical conductivity (**Figure 4**). The number of OTUs was strongest negatively correlated to pH (*r* = -0.65) followed by a slightly weaker correlation to ash concentration (*r* = -0.61) and electrical conductivity (*r* = -0.48). Shannon diversity was strongest negatively correlated to wood ash concentration (*r* = -0.51) followed by a slightly weaker correlation to pH (*r* = -0.50) and a weaker correlation to electrical conductivity (*r* = -0.33) (**Figure 4**).

#### Bacterial Community Composition

**FIGURE 5:**
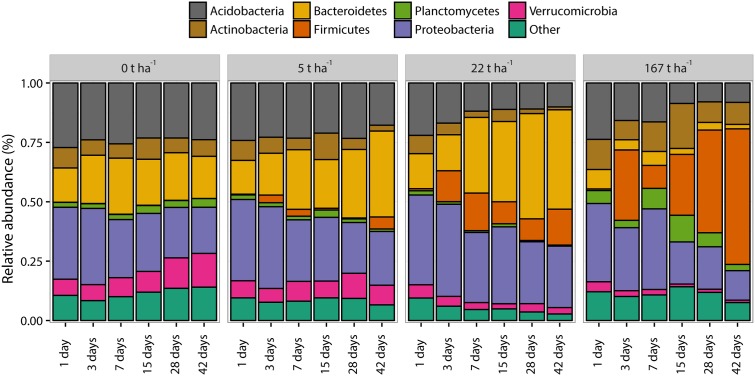
Bacterial community structure at phylum level for increasing wood ash concentrations and increasing incubation time. Presented phyla have an average relative abundance of ≥2%. “Other” represents all phyla with <2% average relative abundance. Each bar represents the mean of triplicates.

A total of 25 bacterial phyla were found in the soil. The bacterial communities were dominated (average relative abundance > 2%) by seven phyla together accounting for 83.7% ± 1.0% (SEM, *n* = 72) of the total relative abundance of the bacterial community (**Figure 5**). The phyla Proteobacteria, Bacteroidetes, and Acidobacteria were the three most dominating phyla in all samples accounting, on average, for 64.1 ± 3.0% of the total relative abundance. The most marked changes in relative abundance (%) were: Acidobacteria decreased from 26.9 ± 0.5% at day 1 with no ash addition to 23.5 ± 0.3%, 17.6 ± 4.2%, 10.1 ± 2.0%, and 8.2 ± 5.7% after 42 days at ash dose 0, 5, 22, and 167 t ha^-1^, respectively. Bacteroidetes increased from 14.2 ± 0.1% at day 1 with no ash addition to 17.3 ± 0.6%, 35.6 ± 5.1%, and 41.4 ± 0.6% after 42 days at ash doses 0, 5, and 22 t ha^-1^, respectively, and a decrease to 1.9 ± 0.9% after 42 days at ash dose 167 t ha^-1^. Firmicutes increased from 0.1 ± 0.01% at day 1 with no ash added to 5.1 ± 3.5%, 15.0 ± 0.2%, and 56.9 ± 23.4% after 42 days at ash doses 5, 22, and 167 t ha^-1^, respectively. The relative abundance of Proteobacteria decreased from day 1 to day 42 for all investigated ash doses with 30.2 ± 0.7% to 19.4 ± 0.1%, 34.0 ± 1.3% to 22.6 ± 0.7%, 37.5 ± 1.0% to 25.9 ± 2.7% and 32.8 ± 1.1% to 12.5 ± 7.1 for ash doses 0, 5, 22, and 167 t ha^-1^, respectively. Supplementary Table 2 shows the relative abundance of the seven most abundant phyla for each dose of ash at days 1 and 42 where the most pronounced differences in the dominant phyla were observed.

**FIGURE 6:**
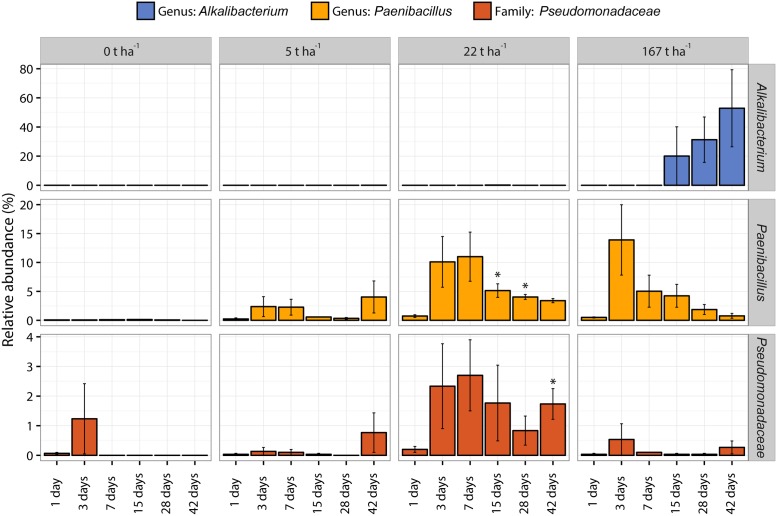
Relative abundance (%) of the genus *Alkalibacterium* (blue bars), genus *Paenibacillus* (yellow bars), and family *Pseudomonadaceae* (red bars) of increasing wood ash addition (from 0 t ha^-1^ in left column to 167 t ha^-1^ in right column) with increasing incubation time on *x*-axis within each plot. Bars represent average of triplicates ± SEM. Asterisks (^∗^) indicate significant (*p* < 0.05) difference in relative abundance to day 1 within the same taxonomic group and ash concentration.

Three taxonomic groups at a lower taxonomic level than phylum (genus: *Alkalibacterium*; genus *Paenibacillus*; family: *Pseudomonadaceae*) show interesting responses in relative abundance after wood ash application and are presented in **Figure 6**. The complete overview of the relative abundance of the whole bacterial community from phyla to genus level is available in Supplementary Data 1.

**FIGURE 7:**
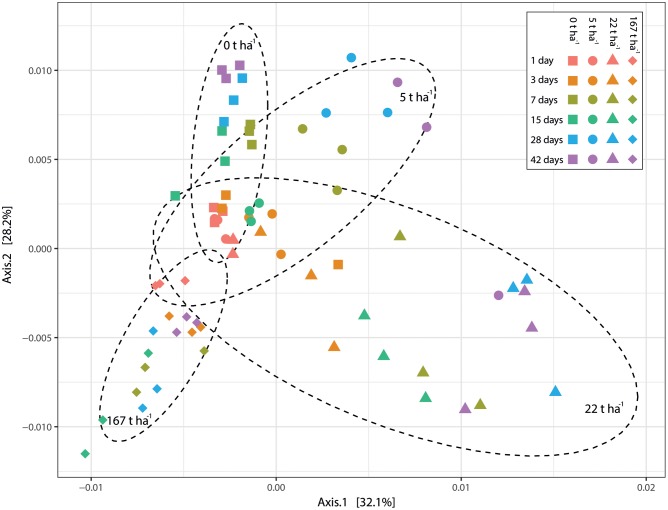
Principal coordinate analysis (PCoA) based on weighted UniFrac dissimilarity. Each point corresponds to a sample. Different colors correspond to different incubation times and different symbols correspond to different wood ash concentrations (as shown in legend). Dashed circles represent 90% confidence ellipse around samples with same wood ash concentration (concentrations presented inside the different ellipses). The percentage of variation explained by the plotted principal coordinates is indicated on the axes.

The added ash doses resulted in significantly (*p* < 0.001, *R*^2^ = 0.276; Adonis (PERMANOVA) on weighted unifrac dissimilarity) different bacterial communities (**Figure 7**). Results from pairwise comparisons between bacterial communities at different ash doses can be seen in Supplementary Table 3. Soil pH, electrical conductivity and incubation time also explained the grouping of the bacterial communities (all *p* < 0.001) but with lower *R*^2^ values (0.243, 0.218, and 0.068 for pH, electrical conductivity and incubation time, respectively) than ash dose.

## Discussion

### Effect of Wood Ash addition on Soil pH and Electrical Conductivity

Soil pH and electrical conductivity correlated significantly with the ash dosage applied to the spruce forest soil. These observations are consistent with previously reported increases in soil pH (Ohno and Susan Erich, 1990; Clapham and Zibilske, 1992; Kahl et al., 1996; Demeyer et al., 2001) and electrical conductivity (Clapham and Zibilske, 1992; Eriksson et al., 1998; Arvidsson and Lundkvist, 2003) in various soil types after ash addition. The increases in soil pH and electrical conductivity were observed 1 day after incubation and remained stable (no significant changes) throughout the incubation period (**Figure 1**). The soil-ash interaction thus reached its equilibrium shortly after application of ash to the soil. These observations are consistent with other studies showing that loose and fine-particulate wood ash is highly reactive in soils (Clapham and Zibilske, 1992; Ulery et al., 1993; Muse and Mitchell, 1995; Vance, 1996; Karltun et al., 2008). However, pH continued to increase during the incubation for the applied ash concentrations 5 and 22 t ha^-1^ with increases from 5.0 to 5.5 and 6.9 to 7.7 from incubation time day 1 to 48 days, respectively.

### Effect of Wood Ash Addition on Culturable Soil Bacteria and Formation of Spores

Measured as CFU, bacteria were stimulated with application of wood ash for all investigated bacterial groups up to an ash dose of 22 t ha^-1^ followed by a significant decrease at 167 t ha^-1^ wood ash addition (**Figure 2**). The increased bacterial numbers after ash application is consistent with previously reported increases in microbial numbers and bacterial growth after ash application (Bååth and Arnebrant, 1994; Fritze et al., 2000; Perkiömäki and Fritze, 2002; Aronsson and Ekelund, 2004). The stimulation of bacterial numbers can to a large extent be explained by the increase in soil pH. The soil pH was raised from acidic (pH 4.3) at an ash dose of 0 t ha^-1^ to around neutral (pH 7.2) at an ash dose of 22 t ha^-1^ which is comparable to studies observing increased bacterial growth rates and numbers in soil with increasing soil pH (Rousk et al., 2009, 2010; Fernández-Calviño et al., 2011). The observed increases in bacterial numbers occurred already 3 days after ash application which most likely is the result of fast growing copiotrophs (*r*-strategist) have favorable conditions in the soil after ash addition. This is supported by the increase in the numbers of *Pseudomonas* up to ash dose 22 t ha^-1^ (**Figure 2C**) because bacteria in the genus *Pseudomonas* are generally thought to be copiotrophic (Smit et al., 2001; Inceoğlu et al., 2011; Lladó and Baldrian, 2017). Better growth of copiotrophic bacteria after wood ash application is probably due to increased nutrient availability. Nutrients are added directly with the wood ash but easily available nutrients are probably also released from lyzed microorganisms which are killed by the ash induced changes to the soil system. Additionally, the increased soil pH has an impact on the fraction of bioavailable nutrients in the soil which potentially improve conditions favoring copiotrophic bacteria (Demeyer et al., 2001).

A wood ash dose of 167 t ha^-1^ resulted in detrimental effects on the soil bacterial numbers with significant decreases. Decreases in bacterial numbers after ash application have, to our knowledge, not been reported before. The 167 t ha^-1^ ash dose is, admittedly, an extreme dose causing extreme changes in the soil system. At this ash dose the soil bacteria are exposed to a soil pH of 11.3 and a 30-fold increase in electrical conductivity compared to the control soil. These extreme conditions are probably very stressful for many soil bacteria adapted to the conditions of the non-manipulated acidic soil. Other ash inherent components such as the high concentration of ions which change soil water osmolarity and heavy metals are also known to be toxic to microorganisms and are possibly contributing to the detrimental effect seen on bacterial numbers at an ash dosage of 167 t ha^-1^.

Numbers of spore forming bacteria were not significantly different from numbers of general bacteria at an ash dose of 167 t ha^-1^ (**Figures 2A,B**). Moreover, the ratio of spore forming bacteria to general bacteria increased markedly from 0.012 to 0.019 at ash doses of 0–22 t ha^-1^ up to 0.26 at an ash dose of 167 t ha^-1^ (Supplementary Figure 1). A large proportion of the bacteria surviving at a ash concentration of 167 t ha^-1^ thus go into a dormant spore stage to withstand the extreme conditions (Rothschild and Mancinelli, 2001). For the ash doses of 0, 5, and 22 t ha^-1^ there were significantly more general bacteria than spore forming bacteria and the ratio of spore forming bacteria to general bacteria was low indicating that bacteria do not need to be in a spore stage to survive the ash induced soil changes here.

### Effects of Wood Ash Addition on Soil Bacterial Community

Richness (observed OTUs) and diversity (Shannon diversity) decreased with increased concentration of wood ash (**Figures 3**, **4**). Richness and diversity showed a clear negative correlation with soil pH, wood ash concentration and electrical conductivity. Of the three variables, pH had the strongest and electrical conductivity the weakest correlation with richness (**Figure 4**). With loss of bacterial richness and diversity the overall genepool of the soil is reduced which potentially reduces soil functioning and the stability of the ecosystem (Wittebolle et al., 2009; Philippot et al., 2013).

However, ANOVA with Tukey *post hoc* test revealed that the decreases in richness and diversity first became significant after 42 days at 167 t ha^-1^ wood ash (**Figure 3**). Wood ash concentrations of 5 and 22 t ha^-1^ did not result in significant differences in richness or diversity during the experimental period. This demonstrates that it indeed requires extreme ash concentrations to provoke a significant impact on the richness and diversity of the bacterial community in this forest soil. Furthermore, as the bacteria seemed to respond gradually during time after ash application, 42 days were needed to observe a significant effect on bacterial richness and diversity.

Addition of wood ash changed the bacterial community composition and the added wood ash doses was the best explanatory variable for the bacterial community groupings. The higher the ash doses added to the soil the more distantly related bacterial communities observed (**Figures 5**, **7** and Supplementary Table 3). Additionally, the variables pH, electrical conductivity and incubation time could also significantly explain the observed grouping of the bacterial communities but with weaker explanatory strength than the wood ash concentration [lower *R*^2^-values using adonis (PERMANOVA)]. Soil pH and electrical conductivity did only show slightly weaker explanatory strengths than wood ash concentration whereas the explanatory strength of time was much weaker. This indicate that the wood ash induced changes in pH and electrical conductivity are important determining variables for the observed changes in the bacterial community composition. Moreover, the results indicate that pH is the more important variable of the two for the observed bacterial community groupings due to a slightly higher explanatory strength of pH in comparison to electrical conductivity. This is consistent with other studies showing pH and electrical conductivity (often used as a measure for salinity) to be determining for soil microbial community structure (Fierer and Jackson, 2006; Fierer et al., 2007; Lauber et al., 2009; Rousk et al., 2010; Kim et al., 2016; Ma et al., 2016). An incubation effect on the bacterial communities was present by the gradual change in bacterial communities of soil incubated without wood ash (**Figure 7**). However, this gradual change over time was also seen in samples with wood ash added and because these samples resulted in significant differences between bacterial communities in comparison to the control soil, the observed bacterial community changes must be regarded mainly as a response to the added wood ash.

The bacterial community composition responses at phylum level (**Figure 5** and Supplementary Table 2) show several trends which can be explained by pH changes and by the ecological classification proposed by Fierer et al. (2007) with copiotrophic (analog to *r*-strategist: fast growing under high nutrient availability, highly variable population size) and oligotrophic (analog to *K*-strategist: slow-growing, more stable population size) bacterial groups, as discussed in the following.

Acidobacteria decreased in relative abundance with wood ash addition. This is similar to previous findings in soil after wood ash application (Noyce et al., 2016). Acidobacteria are predominating in low pH conditions (Rousk et al., 2010; Kielak et al., 2016) and the increase in soil pH after wood ash addition can explain the observed decrease. Furthermore, Acidobacteria are generally oligotrophic (Smit et al., 2001; Fierer et al., 2007; Inceoğlu et al., 2011; Kielak et al., 2016) and the increased competition with copiotrophic bacteria after the addition of wood ash inherent nutrients might also explain the relative decrease of Acidobacteria.

Bacteroidetes increased in relative abundance up to 22 t ha^-1^ wood ash and then decreased with 167 t ha^-1^ wood ash applied. Increase in Bacteroidetes has previously been reported in soil after wood ash application (Noyce et al., 2016). Bacteroidetes has been shown to be positively correlated with soil pH (Lauber et al., 2009; Kim et al., 2016). Additionally, Bacteroidetes exhibits copiotrophic lifestyles (Fierer et al., 2007) and has been shown to be some of the initial metabolizers of labile carbon in soil (Padmanabhan et al., 2003). Wood ash application generally increase the concentration of bioavailable nutrients and dissolved organic carbon (DOC) in soil (Demeyer et al., 2001; Augusto et al., 2008). The observed increase in DOC has been explained by increased mineralization rates. Results of Jokinen et al. (2006) illustrated that microbial activity increased with increasing soil pH (from acidic to neutral) and DOC concentration after wood ash application to soil. The increase in nutrients, the possible increase in easily available carbon and the more neutral pH at ash dose 22 t ha^-1^ gives favorable growth conditions for the copiotrophic Bacteroidetes resulting in a larger fraction of the bacterial community being occupied by this group. Bacteroidetes do not seem to be able to cope with the extreme conditions in the soil after the addition of the high ash dose (167 t ha^-1^) resulting in a drastic decrease in relative abundance of this phylum. Interestingly, *Pseudomonas*, another important copiotrophic group followed the same trend as Bacteroidetes (Smit et al., 2001). This is evident in the CFU result (**Figure 2C**) and in the relative abundance of 16S sequencing results of the *Pseudomonadaceae* (**Figure 6**) with stimulated numbers and relative abundance up to 22 t ha^-1^ wood ash addition followed by a decrease at 167 t ha^-1^ wood ash addition.

The dominant phylum Proteobacteria decreased in relative abundance from day 1 to day 42 for all applied ash doses but no clear trend was found at lower taxonomic levels inspecting the classes of α-, β-, γ-, and δ-Proteobacteria which otherwise have been shown to respond to pH changes (Rousk et al., 2010) and to be copiotrophic bacteria (Fierer et al., 2007). Proteobacteria, however, remained at a high relative abundance (12.5–37.4%) throughout all added ash doses. This might be explained by Proteobacteria in general being rather resistant toward environmental changes (Barnard et al., 2013).

Firmicutes increased in relative abundance with ash addition. This is especially pronounced after 28–42 days at an ash dose of 167 t ha^-1^. Firmicutes are known to be able to cope with various environmental stresses (Barnard et al., 2013) and are further known as endospore forming bacteria (de Hoon et al., 2010). Firmicutes are thus capable of surviving and withstanding the extreme conditions introduced by the concentration of 167 t ha^-1^ wood ash. This is consistent with the CFU results of the present study showing that total number of culturable bacteria is not significantly different from the number of sporeforming bacteria which are present in the soil after 167 t ha^-1^ ash application. The increase in Firmicutes can to a large extent be attributed to the increase of the genus *Alkalibacterium* (**Figure 6**) which dominates the bacterial community after 42 days with 167 t ha^-1^ ash addition. *Alkalibacterium* is known to consist of species that are alkaliphilic and halophilic (Ntougias and Russell, 2001; Nakajima et al., 2005; Ishikawa et al., 2009, 2013). This can explain why this genus survives the extreme increases in pH and salinity (here measured indirectly by electrical conductivity) after the addition of 167 t ha^-1^ wood ash to soil on the expense of other bacterial taxa.

Species within the genus of *Paenibacillus* have many known plant growth promoting capabilities such as N_2_ fixation and suppressing plant pests and pathogens (McSpadden Gardener, 2004; Lal et al., 2016; Rybakova et al., 2016). Responses in a group like *Paenibacillus* can thus indicate changes to the overall soil quality which is highly relevant in a forest plantation like the one investigated in the present study. The presented DNA data allowed us to see changes in this important genus with ash application where we saw an increase in relative abundance at an ash dose of 22 t ha^-1^. Species of *Paenibacillus* have been reported to grow between pH 5.0–12.0, with an optimum at 7.0–7.2 (von der Weid et al., 2002; Smith et al., 2009; Wang et al., 2013). The pH optimum around neutral corresponds well with the observed increases in relative abundance of *Paenibacillus* at an ash dose of 22 t ha^-1^ because pH of the soil is close to the optimum for growth here. The family *Pseudomonadaceae* likewise contains many species important for plant health and soil quality (Martínez-Viveros et al., 2010; Pereg and McMillan, 2015). *Pseudomonadaceae* increase in relative abundance at 22 t ha^-1^ ash addition which is consistent with CFU counts described above.

Increased incubation time amplified the bacterial community responses (**Figures 3**, **5**, **7**). This shows a gradual response of the soil bacteria to the wood ash induced changes in the soil system. In contrast, the culturable bacteria already responded after 3 days of incubation (**Figure 2**). This may be explained by slow degradation of extracellular DNA in soil (Pietramellara et al., 2009) giving a lag in response of the bacterial community as seen through 16S DNA sequencing. If this is true, there might be a more rapid bacterial response to the ash application which we only see gradually as DNA is degraded in soil. Future studies, examining the short-term microbial responses after wood ash application, could circumvent this potential interfering effect by using transcript-based analysis because of RNA’s fast turnover rate in soil.

Our results indicate that wood ash application in concentrations comparable to the upper limits of current legislations in Scandinavian countries (5 t ha^-1^) increased bacterial numbers and increased the relative abundance of copiotrophic bacterial groups on the expense of oligotrophic groups. A similar response was observed for the application of 22 t ha^-1^ but with more pronounced effects than at 5 t ha^-1^. None of these observed bacterial responses at 5–22 t ha^-1^ indicated potential ecosystem damaging effects to the spruce forest plantation investigated here. Detrimental effects on the soil bacteria were, however, observed at a wood ash dose of 167 t ha^-1^ with a significant decrease in diversity and in numbers of culturable bacteria together with predominance of alkaliphilic, halophilic, and sporeforming bacteria. These detrimental effects are likely to cause unwanted effects on the ecosystem functioning. Already 22 t ha^-1^ is an unrealistic ash dosage to apply in forestry but the lack of detrimental effect at this high dose provide a safer ground for concluding that the current allowable wood ash dosages are safe. The observed bacterial responses could be explained by the increase in pH, electrical conductivity and nutrients after wood ash application. The initial pH level of a soil and its buffer capacity and the alkalinity of the applied wood ash is thus determining for potential detrimental effects on soil bacterial communities and thereby ecosystem functioning.

## Conclusion

The results of this study show that wood ash application to forest soil can cause significant changes to bacterial numbers, richness, diversity and community composition. The applied ash doses of 5 and 22 t ha^-1^ increased soil bacterial numbers and resulted in favorable conditions for copiotrophic bacteria and less favorable conditions for oligotrophic bacteria which could be seen directly in a gradually change of the bacterial community composition. Detrimental effects on soil bacteria were only observed in the extreme treatment of 167 t ha^-1^ with decreasing bacterial numbers and a dramatic change of the bacterial community composition. A single genus, known to thrive under alkaline conditions, dominated the bacterial community aligned with the high ash dose (167 t ha^-1^) which did make the soil highly alkaline. Spore forming bacteria represent the majority of the bacteria capable of surviving the high ash dose.

## Author Contributions

TB-A, JTN, JV, JH, RR, RK, HCBH, and CSJ designed the study. TB-A, JTN, JV, and JH did experimental work and data analysis. TB-A wrote the paper in collaboration with all co-authors.

## Conflict of Interest Statement

The authors declare that the research was conducted in the absence of any commercial or financial relationships that could be construed as a potential conflict of interest.
