# Supplementary material for: Wood Ash Induced pH Changes Strongly Affect Soil Bacterial Numbers and Community Composition
Source: Front Microbiol. 2017 Jul 28;8:1400. doi: 10.3389/fmicb.2017.01400 (PMC5532396; doi:10.3389/fmicb.2017.01400)
Supplement: Supplementary file 2 [file Data_Sheet_2.docx]

Supplementary Material

**Wood ash induced pH changes strongly affect soil bacterial numbers and community composition**

Toke Bang-Andreasen^1,2,^ Jeppe Toftgaard Nielsen^3,4^, Jana Voriskova^3,5,6^, Janine Heise^7^, Regin Rønn^2,8,9^, Rasmus Kjøller^2^, Hans Christian Bruun Hansen^4^, Carsten Suhr Jacobsen^1,6*^

^1^ Department of Environmental Science, Aarhus University, DK-4000 Roskilde, Denmark

^2^ Department of Biology, University of Copenhagen, DK-2100 Copenhagen, Denmark

^3^ Department of Geochemistry, Geological Survey of Denmark and Greenland (GEUS), DK-1350 Copenhagen, Denmark

^4^ Department of Plant and Environmental Sciences, University of Copenhagen, DK-1871 Frederiksberg C, Denmark

^5^ Ecology Department, Climate and Ecosystem Sciences, Lawrence Berkeley National Laboratory, Berkeley, CA, USA

^6^ Center for Permafrost (CENPERM), University of Copenhagen, DK-1350 Copenhagen, Denmark

^7^ Section for Geomicrobiology, GFZ German Research Centre for Geosciences, 14473 Potsdam, Germany

^8^ Key Laboratory of Urban Environment and Health, Institute of Urban Environment, Chinese Academy of Sciences, Xiamen, China

^9^ Arctic Station, University of Copenhagen, Qeqertarsuaq, Greenland

*** Correspondence:**Carsten S. Jacobsen
[csj@envs.au.dk](mailto:csj@envs.au.dk)

# Supplementary Information 1:

Calculation of the applied wood ash doses from $\frac{g DW ash}{g DW soil}$ into $\frac{tonnes ash}{hectare soil}$.

Bulk density of the soil: 0.65 g / cm^3^

Depth of where ash is reactive in soil column: 3 cm

Gravimetric water content of the soil: 1.45 g water / g dry weight soil

The following equation was used which includes all units listed above:

$$\frac{\frac{g DW ash}{g DW soil}\cdot{0.65\frac{g}{{cm}^{3}}\cdot3 cm\cdot10}^{8}\frac{{cm}^{2}}{hectare}}{{10}^{6}\frac{g}{ton}}\cdot\frac{1}{1+1.45\frac{g water}{g DW soil}}=\frac{tonnes}{hectare}$$

# Supplementary Figure 1


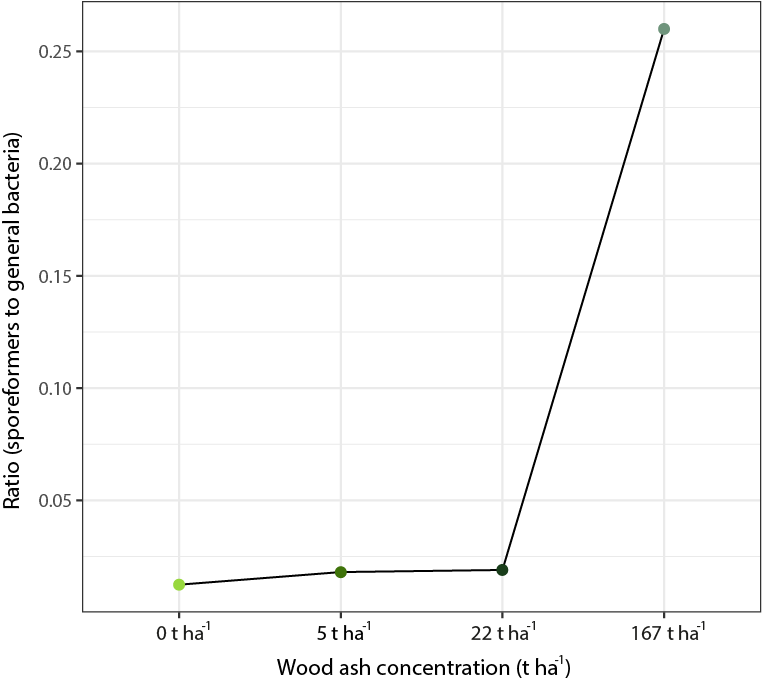


**Supplementary Figure 1.** Ratio of spore forming bacteria to general bacteria in increasing wood ash concentrations. Ratios are calculated from mean values of colony forming units as shown in figure 2.

# Supplementary Table 1:

Nucleotide sequences of primers used for library preparation.

| Forward primers | Tag | Linker | Template specific primer | Final sequence |
| --- | --- | --- | --- | --- |
| 515F_01 | ACGTA | GT | GTGCCAGCMGCCGCGGTAA | ACGTAGTGTGCCAGCMGCCGCGGTAA |
| 515F_02 | TATG | GT | GTGCCAGCMGCCGCGGTAA | TATGGTGTGCCAGCMGCCGCGGTAA |
| 515F_03 | ACACG | GT | GTGCCAGCMGCCGCGGTAA | ACACGGTGTGCCAGCMGCCGCGGTAA |
| 515F_04 | GTAG | GT | GTGCCAGCMGCCGCGGTAA | GTAGGTGTGCCAGCMGCCGCGGTAA |
| 515F_05 | CTGATA | GT | GTGCCAGCMGCCGCGGTAA | CTGATAGTGTGCCAGCMGCCGCGGTAA |
| 515F_06 | CGATAC | GT | GTGCCAGCMGCCGCGGTAA | CGATACGTGTGCCAGCMGCCGCGGTAA |
| 515F_07 | TGCT | GT | GTGCCAGCMGCCGCGGTAA | TGCTGTGTGCCAGCMGCCGCGGTAA |
| 515F_08 | CGCAGA | GT | GTGCCAGCMGCCGCGGTAA | CGCAGAGTGTGCCAGCMGCCGCGGTAA |
| 515F_09 | GACGCA | GT | GTGCCAGCMGCCGCGGTAA | GACGCAGTGTGCCAGCMGCCGCGGTAA |
| 515F_10 | AGAT | GT | GTGCCAGCMGCCGCGGTAA | AGATGTGTGCCAGCMGCCGCGGTAA |
| 515F_11 | ACTACA | GT | GTGCCAGCMGCCGCGGTAA | ACTACAGTGTGCCAGCMGCCGCGGTAA |
| 515F_12 | GATCT | GT | GTGCCAGCMGCCGCGGTAA | GATCTGTGTGCCAGCMGCCGCGGTAA |
|  |  |  |  |  |
| Reverse primers | Tag | Linker | Template specific primer | Final sequence |
| 806R_01 | ACGTA | CC | GGACTACHVGGGTWTCTAAT | ACGTACCGGACTACHVGGGTWTCTAAT |
| 806R_02 | TATG | CC | GGACTACHVGGGTWTCTAAT | TATGCCGGACTACHVGGGTWTCTAAT |
| 806R_03 | ACACG | CC | GGACTACHVGGGTWTCTAAT | ACACGCCGGACTACHVGGGTWTCTAAT |
| 806R_04 | GTAG | CC | GGACTACHVGGGTWTCTAAT | GTAGCCGGACTACHVGGGTWTCTAAT |
| 806R_05 | CTGATA | CC | GGACTACHVGGGTWTCTAAT | CTGATACCGGACTACHVGGGTWTCTAAT |
| 806R_06 | CGATAC | CC | GGACTACHVGGGTWTCTAAT | CGATACCCGGACTACHVGGGTWTCTAAT |
| 806R_07 | TGCT | CC | GGACTACHVGGGTWTCTAAT | TGCTCCGGACTACHVGGGTWTCTAAT |
| 806R_08 | CGCAGA | CC | GGACTACHVGGGTWTCTAAT | CGCAGACCGGACTACHVGGGTWTCTAAT |
| 806R_09 | GACGCA | CC | GGACTACHVGGGTWTCTAAT | GACGCACCGGACTACHVGGGTWTCTAAT |
| 806R_10 | AGAT | CC | GGACTACHVGGGTWTCTAAT | AGATCCGGACTACHVGGGTWTCTAAT |
| 806R_11 | ACTACA | CC | GGACTACHVGGGTWTCTAAT | ACTACACCGGACTACHVGGGTWTCTAAT |
| 806R_12 | GATCT | CC | GGACTACHVGGGTWTCTAAT | GATCTCCGGACTACHVGGGTWTCTAAT |

# Supplementary Table 2

Relative abundance (%) of the seven most abundant phyla identified in the soil of the microcosms for the wood ash doses of 0, 5, 22 and 167 t ha^-1^ at day 1 and 42. Numbers are the average of triplicates ± SEM. Numbers in bold indicate significant difference (*p*<0.05) between the relative abundance at day 1 and 42. Using the software STAMP, White’s non-parametric t-test with Benjamini-Hochberg FDR correction was used to test for significant differences. Day 1 and 42 were chosen because the most different bacterial communities in general are observed between these incubation times.

|  | Relative abundance (%) | | | | | | | |
| --- | --- | --- | --- | --- | --- | --- | --- | --- |
|  | 0 t ha^-1^ | | 5 t ha^-1^ | | 22 t ha^-1^ | | 167 t ha^-1^ | |
| Phylum | Day 1 | Day 42 | Day 1 | Day 42 | Day 1 | Day 42 | Day 1 | Day 42 |
| *Acidobacteria* | 26.9 | 23.5 | 24.0 | 17.6 | **21.9** | **10.1** | 23.5 | 8.2 |
|  | ± 0.5 | ± 0.3 | ± 0.7 | ± 4.2 | **± 0.5** | **± 2.0** | ± 1.3 | ± 5.7 |
| *Actinobacteria* | **8.6** | **6.9** | **8.4** | **2.5** | **7.7** | **1.2** | 12.6 | 9.3 |
|  | **± 0.2** | **± 0.1** | **± 0.1** | **± 0.7** | **± 0.3** | **± 0.1** | ± 1.1 | ± 5.2 |
| *Bacteriodetes* | 14.2 | 17.3 | 14.0 | 35.6 | **14.5** | **41.4** | **8.1** | **1.9** |
|  | ± 0.1 | ± 0.6 | ± 0.2 | ± 5.1 | **± 0.9** | **± 0.6** | **± 0.8** | **± 0.9** |
| *Firmicutes* | 0.1 | 0.1 | 0.3 | 5.1 | **0.9** | **15.0** | 0.7 | 56.9 |
|  | ± 0.01 | ± 0.01 | ± 0.2 | ± 3.5 | **± 0.3** | **± 0.2** | ± 0.1 | ± 23.4 |
| *Planctomycetes* | **2.1** | **3.6** | 1.9 | 1.1 | **1.8** | **0.4** | 5.4 | 2.7% |
|  | **± 0.1** | **± 0.1** | ± 0.1 | ± 0.3 | **± 0.1** | **± 0.1** | ± 0.7 | ± 0.2 |
| *Proteobacteria* | **30.2** | **19.4** | **34.0** | **22.6** | **37.5** | **25.9** | 32.8 | 12.5 |
|  | **± 0.7** | **± 0.1** | **± 1.3** | **± 0.7** | **± 1.0** | **± 2.7** | ± 1.1 | ± 7.1 |
| *Verrumicrobia* | **6.8** | **14.1** | 7.2 | 8.27 | 5.6 | 2.8 | **4.2** | **1.0** |
|  | **± 0.4** | **± 0.1** | ± 0.4 | ± 2.0 | ± 0.2 | ± 0.8 | **± 0.1** | **± 0.6** |

# Supplementary Table 3

Pairwise testing for significant difference in bacterial community between increasing wood ash concentrations. Statistical test used: Adonis (PERMANOVA).

|  | 7 % | 28 % | 210 % |
| --- | --- | --- | --- |
| 0 % | P < 0.001  R^2^ = 0.132 | P < 0.001  R^2^ = 0.366 | P < 0.001  R^2^ = 0.458 |
| 7 % |  | P < 0.001  R^2^ = 0.215 | P < 0.001  R^2^ = 0.447 |
| 28 % |  |  | P < 0.001  R^2^ = 0.405 |
